# Data-Aided Maximum Likelihood Joint Angle and Delay Estimator Over Orthogonal Frequency Division Multiplex Single-Input Multiple-Output Channels Based on New Gray Wolf Optimization Embedding Importance Sampling

**DOI:** 10.3390/s24175821

**Published:** 2024-09-07

**Authors:** Maha Abdelkhalek, Souheib Ben Amor, Sofiène Affes

**Affiliations:** 1 The Wireless Lab, EMT Centre, Institut National de la Recherche Scientifique (INRS), Montreal, QC H5A 1K6, Canada; souheib.ben_amor@nokia.com (S.B.A.); sofiene.affes@inrs.ca (S.A.); 2 Nokia, 90120 Oulu, Finland

**Keywords:** importance sampling (IS), gray wolf optimization (GWO), data-aided (DA), joint angle and delay estimation (JADE), maximum likelihood (ML), multi-carrier, orthogonal frequency division multiplex (OFDM), multi-path, single-input multiple-output (SIMO)

## Abstract

In this paper, we propose a new data-aided (DA) joint angle and delay (JADE) maximum likelihood (ML) estimator. The latter consists of a substantially modified and, hence, significantly improved gray wolf optimization (GWO) technique by fully integrating and embedding within it the powerful importance sampling (IS) concept. This new approach, referred to hereafter as GWOEIS (for “GWO embedding IS”), guarantees global optimality, and offers higher resolution capabilities over orthogonal frequency division multiplex (OFDM) (i.e., multi-carrier and multi-path) single-input multiple-output (SIMO) channels. The traditional GWO randomly initializes the wolfs’ positions (angles and delays) and, hence, requires larger packs and longer hunting (iterations) to catch the prey, i.e., find the correct angles of arrival (AoAs) and time delays (TDs), thereby affecting its search efficiency, whereas GWOEIS ensures faster convergence by providing reliable initial estimates based on a simplified importance function. More importantly, and beyond simple initialization of GWO with IS (coined as IS-GWO hereafter), we modify and dynamically update the conventional simple expression for the convergence factor of the GWO algorithm that entirely drives its hunting and tracking mechanisms by accounting for new cumulative distribution functions (CDFs) derived from the IS technique. Simulations unequivocally confirm these significant benefits in terms of increased accuracy and speed Moreover, GWOEIS reaches the Cramér–Rao lower bound (CRLB), even at low SNR levels.

## 1. Introduction

JADE (For the reader’s convenience, please find and refer to the full list of all abbreviations adopted in this paper, at the very end, right before the bibliography section) is a crucial operation in many digital receivers. Highly accurate and computationally inexpensive JADE is required in many fields ranging from military applications such as RADAR or SONAR systems to wireless indoor positioning [1,2] and wireless communication systems [3]. Moreover, the estimation of AoAs and TDs enable the design of highly accurate localization techniques [4]. Many existing works have focused on solving the JADE problem. Most fall under the subspace-based category such as Multiple Signal Classification (MUSIC) [5], ESPRIT [6], and the 2D unitary matrix pencil (UMP) [7]. One of the iterative ML estimators based on the space-alternating generalized expectation maximization (SAGE) algorithm was proposed in [8]. The approach in [9] mainly targets only the DOA estimation. It introduces a DOA estimation algorithm in a full-dimension MIMO system by solving the maximum likelihood estimation using expectation-maximization (EM) algorithm. Recently, a tensor-based approach for channel and target parameter estimation was proposed in [10]. Also, a non-iterative ML estimator that tackles the JADE problem in a multi-carrier transmission context was developed in [11] using the IS technique. More recently, we proposed a new ML JADE solution in a non-DA (NDA) single-carrier scenario [12]. Shortly after, we have tackled the same scenario by making IS initialize the differential evolution (DE) technique in [13]. Referred to hereafter as IS-DE, it was a first attempt to tackle JADE by exploiting a bio-inspired optimization approach.

Under this category, GWO has notably been used to optimize and solve many engineering problems. The best-known applications are numerical simulations and stability fields [14,15], feature acquisition selection, dataset classification, neural networks training [16], etc. A multi-objective GWO was developed for cloud computing in [17], and for wireless sensor networks in [18]. It was adapted to a multi-robot application in [19], and to unmanned aerial vehicles (UAVs) in [20]. A GWO-based optimal channel estimation technique was proposed for large-scale MIMO in LTE networks in [21]. A dragonfly-evaluated gray wolf optimization (DA-GWO) model was introduced in [22], which hybridizes the concepts of dragonfly algorithm and GWO for channel estimation in millimeter wave massive MIMO system. In [23], we find also that the GWO algorithm was used only for direction of arrival (DoA) estimation.

Nevertheless, GWO suffers from slow convergence, limited solution accuracy, and susceptibility to getting trapped in local optima. Many improvements to GWO were proposed in different applications [24,25,26,27], but none were to tackle JADE, to the best of our knowledge.

In this paper, we exploit GWO [28] to solve JADE over OFDM SIMO transmissions in multi-path environments. The main idea consists of improving the GWO by initializing the wolf positions using the IS technique instead of random positions. More importantly, and beyond simple initialization of GWO with IS (coined as IS-GWO hereafter), we modify and dynamically update the conventional simple expression for the convergence factor of the GWO algorithm that entirely drives its hunting and tracking mechanisms by accounting for new CDFs derived from the IS technique. Numerical assessments will confirm the advantages of the proposed GWOEIS over IS, GWO, IS-GWO, DE, IS-DE, and other state-of-the-art JADE solutions in terms of estimation accuracy, population or sample size (e.g., number of wolves), global convergence, and convergence speed. The remainder of this paper is organized as follows. Section 2 introduces the multi-carrier SIMO system model in multi-path environments. Section 3 addresses JADE, first by deriving the concentrated likelihood function (CLF), then the IS technique, and ultimately the main common algorithmic steps and the key variations we introduced to some that ultimately encompass GWO, IS-GWO, and the proposed GWOEIS. Section 4 discusses our computer simulations and results, whereas Section 5 concludes our work.

The adopted notations are as follows. Vectors and matrices are represented in lower- and upper-case bold fonts, respectively. Moreover, ${\left\{.\right\}}^{T}$ and ${\left\{.\right\}}^{H}$ denote the conjugate and Hermitian (i.e., transpose conjugate) operators. The Euclidean norm of any vector is denoted as ||.||, and $I_{N}$ denotes the (N×N) identity matrix. For any matrix X, ${\left[X\right]}_{l}$, and ${\left[X\right]}_{p,l}$, denote its *l*th column and (p,l)th entry, respectively. The kronecker product of any two matrices X and Y is denoted as X⊗Y. For any vector x, ${\left[x\right]}_{p}$ denotes its *p*th entry or element, and diag{x} refers to the diagonal matrix whose elements are those of x. Moreover, |.| returns the modulus of any complex number. Finally, *j* is the pure imaginary number (i.e., $j^{2}=-1$), and the notation ≜ is used for definitions.

## 2. System Model

We consider a SIMO OFDM system characterized by a single transmitting and *P* receiving antenna elements and *K* sub-carriers. At each time period, this system transmits over these sub-carriers *K* symbols $s={\left[s_{1},s_{2},\ldots ,s_{K}\right]}^{T}$, all belonging to an *M*-ary constellation alphabet $C^{M}$, and are assumed to be known at pilot transmission periods (i.e., replaced a priori by 1) in the DA-type estimation scheme we are adopting here. The transmit data then goes through a multi-path channel consisting of different paths, whose number *Q* is assumed to be known.

The resulting multi-path SIMO channel is characterized by *Q* different AoAs $\theta =\left[\theta_{1},\theta_{2},\cdots ,\theta_{Q}\right]\in U{\left([\frac{-\pi }{2},\frac{\pi }{2}]\right)}^{Q}$; *Q* different TDs $\tau =\left[\tau_{1},\tau_{2},\cdots ,\tau_{Q}\right]\in U{\left([0,\tau_{\max}]\right)}^{Q}$, where $\tau_{\max}$ can be chosen to be as large as desired; $U\left([v_{\min},v_{\max}]\right)$ denotes a uniform distribution over the interval $\left[v_{\min},v_{\max}\right]$; and *Q* complex gains $\gamma ={\left[\gamma_{1},\gamma_{2},\cdots ,\gamma_{Q}\right]}^{T}$. All these three parameter vectors are assumed to be unknown.

At the receiver side, the observation signal, $x_{p}\left(k\right)$, over the *p*th antenna and the *k*th sub-carrier, is given by:

(1) $$x_{p}\left(k\right)=h_{p}\left(k\right)s_{k}+n_{p}\left(k\right),$$

where $n_{p}\left(k\right)$ is an additive white Gaussian noise (AWGN) with zero mean and variance $\sigma^{2}$, and $h_{p}\left(k\right)$ is the channel frequency response (CFR), defined as follows:

(2) $$h_{p}\left(k\right)=\sum_{q=1}^{Q}\gamma_{q}e^{-j2\pi k\Delta_{f}\tau_{q}}e^{-j2\pi p\sin(\theta_{q})},$$

where $\Delta_{f}$ is the sub-carrier spacing. By stacking, the scalar signal observation in (1) received at each *k*th sub-carrier into a single observation vector $x\left(k\right)={\left[x_{1}\left(k\right),x_{2}\left(k\right),\cdots ,x_{P}\left(k\right)\right]}^{T}$ is modeled as follows:

(3) $$x\left(k\right)=h\left(k\right)s_{k}+n\left(k\right),$$

where $n\left(k\right)={\left[n_{1}\left(k\right),n_{2}\left(k\right),\ldots ,n_{P}\left(k\right)\right]}^{T}$ is an i.i.d. and spatially uncorrelated zero-mean Gaussian noise vector and h(k) is the P×1 CFR vector over all antennas, defined as:

(4) $$\begin{array}{ccc} h(k) & = & \sum_{q=1}^{Q}a\left(\theta_{q}\right)\gamma_{q}e^{-j2\pi k\Delta_{f}\tau_{q}} \end{array}$$

(5) $$\begin{array}{ccc} & = & \bar{A}\left(\theta \right)D_{k}\left(\tau \right)\gamma , \end{array}$$

where $\bar{A}\left(\theta \right)≜\left[a\left(\theta_{1}\right),a\left(\theta_{2}\right),\ldots ,a\left(\theta_{Q}\right)\right]$ is a P×Q steering matrix, a(θ) is the P×1 steering vector at AoA θ defined for simplicity and without loss of generality here over a uniform linear array (ULA) as:

$$\begin{array}{c} a\left(\theta \right)\;≜\;{\left[1,\;e^{-j\pi \sin(\theta )},\cdots ,\;e^{-j(P-1)\pi \sin(\theta )}\right]}^{T}, \end{array}$$

and $D_{k}\left(\tau \right)\;≜\mathrm{diag}\left(e^{-j2\pi \bar{k}\Delta_{f}\tau_{1}},e^{-j2\pi \bar{k}\Delta_{f}\tau_{2}},\cdots ,e^{-j2\pi \bar{k}\Delta_{f}\tau_{Q}}\right)$ is the Q×Q TDs matrix, in which $\bar{k}$ is some index translation of k=1,⋯,K (e.g., $\bar{k}=k-1$, $\bar{k}=k-K/2-1$ if *K* is even, $\bar{k}=k-\left(K-1\right)/2$ if *K* is odd, etc.), with no impact at all on what follows.

For an even more compact notation, we now stack the P×1 CFR vector h(k) over all sub-carriers to obtain the KP×1 total CFR vector H as follows:

(6) $$H={\left[\ldots ,h{\left(k\right)}^{T},\ldots \right]}^{T}=A\left(\theta \right)D\left(\tau \right)\gamma ,$$

where A(θ) is the KP×KQ steering matrix defined as:

(7) $$A\left(\theta \right)=\mathrm{diag}\;\left\{\bar{A}\left(\theta \right),\bar{A}\left(\theta \right),\cdots ,\bar{A}\left(\theta \right)\right\},\;\left(Ktimes\right)$$

and D(τ) is the KQ×Q TDs matrix defined as:

(8) $$D\left(\tau \right)≜{\left[D_{1}{\left(\tau \right)}^{T}D_{2}{\left(\tau \right)}^{T}\cdots D_{K}{\left(\tau \right)}^{T}\right]}^{T}.$$

Hence, at pilot period transmissions where sub-carriers are not modulated (i.e., $s_{k}=1$), after we stack both x(k) and n(k) in the same way we did to transform h(k) into H, we obtain:

(9) X=H+N.

## 3. Joint Angle and Delay Estimation (JADE)

### 3.1. Derivation of the CLF

At any given pilot transmission period, we can derive the log-likelihood function (LLF) that depends on all three unknown parameter vectors θ, τ, and γ as follows:

(10) $$\begin{array}{ccc} L\left(\theta ,\tau ,\gamma \right) & = & ||X-H|{|}^{2}=||X-A\left(\theta \right)D\left(\tau \right)\gamma |{|}^{2}. \end{array}$$

Hence, we can estimate γ using the least squares (LS) solution as follows:

(11) $$\begin{array}{ccc} {\hat{\gamma }}_{\mathrm{LS}} & = & {\left[A(\theta )D(\tau )\right]}^{†}x=B^{†}x, \end{array}$$

where $B^{†}={\left(B^{H}B\right)}^{-1}B$ is the Moore–Penrose pseudo-inverse of B. By injecting ${\hat{\gamma }}_{\mathrm{LS}}$ into (10), we obtain the CLF:

(12) $$F_{c}\left(\theta ,\tau \right)=x^{H}B{\left(B^{H}B\right)}^{-1}B^{H}x.$$

Hence, we can obtain the joint ML estimates of θ and τ as the optimal solution $\Xi_{opt}$ to the following optimization problem:

(13) $$\Xi_{opt}≜\left[\theta_{opt},\;\tau_{opt}\right]=\underset{\Xi ≜[\theta ,\tau ]}{\mathrm{argmax}}\;\left(F_{c}\left(\theta ,\tau \right)\right).$$

### 3.2. Overview/Summary of IS for ML DA JADE

We start by approximating $B^{H}B$ in (12) as follows:

(14) $$\begin{array}{c} B^{H}B\approx P\;K\;I_{Q}, \end{array}$$

where $I_{Q}$ denotes the (Q×Q) identity matrix. Then, we plug (14) into (12) to obtain:

(15) $$\begin{array}{ccc} \;\;F_{c}\left(\theta ,\tau \right)\;\;\;\; & \approx & \;\;\;\;\frac{1}{PK}\;x^{H}BB^{H}x\approx \frac{1}{PK}\left|\right|B^{H}x{\left|\right|}^{2},\\ & \approx & \;\;\;\;\frac{1}{PK}\sum_{q=1}^{Q}|\;\sum_{k=1}^{K}{\left[\bar{A}\left(\theta \right)D_{k}\left(\tau \right)\right]}^{H}\;x\left(k\right){|}^{2}\;. \end{array}$$

Now, injecting the expressions of $\bar{A}\left(\theta \right)$ and $D_{k}\left(\tau \right)$ into (15), we obtain the approximate CLF:

(16) $$F_{c}\left(\theta ,\tau \right)\approx \frac{1}{PK}\sum_{q=1}^{Q}\psi \left(\theta_{q},\tau_{q}\right),$$

where ψ(θ,τ) is the so-called periodogram of the observation signal [11] given by:

(17) $$\begin{array}{ccc} \;\;\;\;\;\;\;\;\;\;\;\;\psi (\;\theta ,\tau \;) & = & |\sum_{p=1}^{P}\sum_{k=1}^{K}\;e^{-j\pi (p-1)\sin\left(\theta \right)}e^{-j2\pi \bar{k}\Delta f\tau }x_{p}^{*}\left(k\right){|}^{2}. \end{array}$$

Relying on the observations made above, we summarize the IS process in [11] of generating the *R* realizations of the AoA-TD couples according to the following steps:

**Step (1):** we start by evaluating the periodogram $\psi (\theta_{i},\tau_{j})$ in (17) at all grid points $\left(\theta_{i},\tau_{j}\right)\in \Gamma \left(\frac{-\pi }{2},\frac{\pi }{2},\delta_{\theta }\right)\times \Gamma \left(0,\tau_{\max},\delta_{\tau }\right)$ where $\Lambda (v_{\min},v_{\max},\delta_{v})$ denotes the set of points obtained over the interval $\left[v_{\min},v_{\max}\right]$ with a uniform sampling step $\delta_{v}$.
**Step (2):** we evaluate the so-called joint pseudo-pdfs [11] set of values over the above AoA-TD grid as follows:
  (18) $$\begin{array}{c} \bar{\Phi }\left(\theta_{i},\tau_{j}\right)=\frac{\exp\left\{\rho_{1}\psi \left(\theta_{i},\tau_{j}\right)\right\}}{\sum_{i}\sum_{j}\exp\left\{\rho_{1}\psi \left(\theta_{i},\tau_{j}\right)\right\}\delta_{\theta }\delta_{\tau }}, \end{array}$$
  where $\rho_{1}$ is a design parameter to be chosen properly later on.
**Step (3):** we evaluate the marginal pseudo-pdf of $\tau_{j}$ as follows:
  (19) $$\begin{array}{c} {\bar{\phi }}_{\tau }\left(\tau_{j}\right)=\sum_{i}{\bar{\phi }}_{\theta ,\tau }\left(\theta_{i},\tau_{j}\right)\delta_{\theta }. \end{array}$$

Then, we can find the initial TD estimates that correspond to the *Q* maxima of (19) as:

(20) $$\begin{array}{c} \left[{\hat{\tau }}_{1}^{0},{\hat{\tau }}_{2}^{0},\cdots ,{\hat{\tau }}_{Q}^{0}\right]=\underset{\tau }{\mathrm{argmax}}{|}_{Q}\left({\bar{\phi }}_{\tau }\left(\tau \right)\right), \end{array}$$

where ${\mathrm{argmax}|}_{Q}\left(f\right)$ returns the *Q* maxima of the function *f*.

**Step (4):** for q=1,2,⋯,Q, we compute the pseudo-CDF of $\tau_{j}$ as follows:
  (21) $$\begin{array}{c} J_{\tau_{q}}\left(\tau_{j}\right)=\sum_{l\leq j}{\bar{\phi }}_{\tau_{q}}\left(\tau_{l}\right)\delta_{\tau }\;\;\;\;\forall \;\tau_{l}\;\in \lambda_{{\hat{\tau }}_{q}^{0}}, \end{array}$$
  where
  (22) $$\lambda_{{\hat{\tau }}_{q}^{0}}=\Gamma \left({\hat{\tau }}_{q}^{0}-\Delta_{\tau },{\hat{\tau }}_{q}^{0}+\Delta_{\tau },\delta_{\tau }\right).$$
**Step (5):** For q=1,2,⋯,Q, we generate *R* realizations ${\left\{u_{q}^{\left(r\right)}∼U\left([0,1]\right)\right\}}_{r=1}^{R}$. Then, we apply a linear interpolation to obtain the *r*th TD realization:
  (23) $${\hat{\tau }}_{q}^{\left(r\right)}=J_{\tau_{q}}^{-1}\left(u_{q}^{\left(r\right)}\right).$$
**Step (6):** We evaluate the conditional pseudo-pdf of θ given $\tau ={\hat{\tau }}_{q}^{0}$ for q=1,2,⋯,Q as follows:
  (24) $$\begin{array}{c} {\bar{\phi }}_{\theta |\tau }\left(\theta_{i}|{\hat{\tau }}_{q}^{0}\right)=\frac{{\bar{\phi }}_{\theta ,\tau }\left(\theta_{i},{\hat{\tau }}_{q}^{0}\right)}{{\bar{\phi }}_{\tau }\left({\hat{\tau }}_{q}^{0}\right)}. \end{array}$$

Then, we obtain the initial *Q* AoA estimates as:

(25) $$\begin{array}{c} {\hat{\theta }}_{q}^{0}=\underset{\theta }{\mathrm{argmax}}\left({\bar{\phi }}_{\theta |\tau }\left(\theta |{\hat{\tau }}_{q}^{0}\right)\right)\;for\;q=1,\cdots ,Q. \end{array}$$

**Step (7):** similarly to Step 4, we compute the conditional pseudo-CDF as:
  (26) $$\begin{array}{c} J_{\theta_{q}|\tau_{q}}\left(\theta_{j}|{\hat{\tau }}_{q}^{\left(r\right)}\right)=\sum_{l\leq j}{\bar{\phi }}_{\theta |\tau }\left(\theta_{i}|{\hat{\tau }}_{q}^{\left(r\right)}\right)\delta_{\tau }\;\;\forall \;\theta_{l}\;\in \lambda_{{\hat{\theta }}_{q}^{0}}. \end{array}$$
  where
  (27) $$\lambda_{{\hat{\theta }}_{q}^{0}}=\Gamma \left({\hat{\theta }}_{q}^{0}-\Delta_{\theta },{\hat{\theta }}_{q}^{0}+\Delta_{\theta },\delta_{\theta }\right).$$
**Step (8):** Similarly to Step 5, we generate *R* realizations ${\left\{u_{q}^{\left(r\right)}∼U\left([0,1]\right)\right\}}_{r=1}^{R}$ for q=1,2,⋯,Q. Then, we apply a linear interpolation to obtain the *r*th AoA realization:
  (28) $${\hat{\theta }}_{q}^{\left(r\right)}=J_{\theta_{q}|\tau_{q}}^{-1}\left(u_{q}^{\left(r\right)}\right).$$

Thus, we are able to generate *R* JADE realizations as:

(29) $${\left\{{\hat{\theta }}^{\left(r\right)}\right\}}_{r=1}^{R}\;\mathrm{and}\;{\left\{{\hat{\tau }}^{\left(r\right)}\right\}}_{r=1}^{R},$$

where ${\hat{\theta }}^{\left(r\right)}=\left[{\hat{\theta }}_{1}^{\left(r\right)},{\hat{\theta }}_{2}^{\left(r\right)},\cdots ,{\hat{\theta }}_{Q}^{\left(r\right)}\right]$ is the AoAs vector estimate, and ${\hat{\tau }}^{\left(r\right)}=\left[{\hat{\tau }}_{1}^{\left(r\right)},{\hat{\tau }}_{2}^{\left(r\right)},\cdots ,{\hat{\tau }}_{Q}^{\left(r\right)}\right]$ is the TDs vector estimate. These realizations readily enable the direct implementation of an IS ML solution to (13) as:

(30) $${\hat{\Xi }}_{IS}=\left[{\hat{\theta }}_{IS}=\frac{\sum_{r=1}^{R}{\hat{\theta }}^{\left(r\right)}}{R},\;\;{\hat{\tau }}_{IS}=\frac{\sum_{r=1}^{R}{\hat{\tau }}^{\left(r\right)}}{R}\right].$$

### 3.3. GWO vs. Combining|Embedding IS (IS-GWO|GWOEIS)

GWO is inspired by the leadership hierarchy and the hunting mechanism of gray wolves in nature [28]. In a pack or population of, say, $R^{\prime }$ individuals, we identify four types of gray wolves that emulate their leadership hierarchy: the α-type are responsible for making hunting decisions (representing the solutions with best results). The β-type help the α-type in decision making and act as their best substitute-candidates when one of them becomes old or dies (second-best solutions in the population). The δ-type have only to submit to the α- and β-types (third-best solutions). And the ω-type are of the lowest rank, and must yield to the dominant ones. Guided by this “social” hierarchy’s rules, gray wolves proceed to hunt along three main and consecutive stages: (1) tracking, chasing, and approaching the prey; (2) pursuing, encircling, and harassing it once it stops moving; and (3) attacking it.

Mathematically and generally speaking, GWO translates the leadership hierarchy and the hunting mechanism summarily described above due to lack of space into an optimization by search (i.e., hunting) in any multi-dimensional space whose best solution (i.e., prey) that minimizes a given criterion (i.e., so-called “fitness function”) is found in an iterative manner by mimicking the gray wolves (i.e., search agents) hunting behavior (i.e., search adaptation rules).

In the present case, $\Xi_{opt}≜\left[\theta_{opt},\;\tau_{opt}\right]$ and $-F_{c}\left(\theta ,\tau \right)$ in (13) stand, respectively, for the prey to be hunted and the fitness function to be minimized in a 2Q-dimensional space. Hence, GWO translates as follows:

**Step (1):** first, the wolves’ positions are initialized in the 2Q space according to one of the following cases (a) or (b).
**Step (1.a) [GWO]:** The conventional GWO initially places the wolves pack of $R^{\prime }=R$ individuals at random positions in the 2Q search space ${\left\{\Sigma_{0}^{\left(r\right)}\right\}}_{r=1}^{R}=\left\{\{\Sigma_{\theta }^{\left(r\right)},\;\Sigma_{\tau }^{\left(r\right)}\right\}{\}}_{=1}^{R}$ where $\Sigma_{\theta }^{\left(r\right)}={\left\{U\left([-\frac{\pi }{2},\frac{\pi }{2}\right)\right\}}_{q=1}^{Q}$ and $\Sigma_{\tau }^{\left(r\right)}={\left\{U\left([0,\tau_{\max}]\right)\right\}}_{q=1}^{Q}$. Hence, it requires larger packs and longer hunting (iterations) to catch the prey, i.e., find the correct angles of arrival (AoAs) and time delays (TDs) without guaranteeing global convergence.
**Step (1.b) [IS-GWO or GWOEIS]:** Instead of random initial placement, IS-GWO and GWOEIS position the wolves at ${\left\{\Sigma_{0}^{\left(r\right)}\right\}}_{r=1}^{R}={\left\{\zeta^{\left(r\right)}=\left\{\theta_{1}^{\left(r\right)},\cdots ,\theta_{Q}^{\left(r\right)},\tau_{1}^{\left(r\right)},\cdots ,\tau_{Q}^{\left(r\right)}\right\}\right\}}_{r=1}^{R}$, stemming from the $R^{\prime }=R$ realizations generated in (29). Hence, even with relatively less realizations, this still guarantees global convergence, and it would always provide good-enough rough initialization values to GWOEIS to make the latter converge much faster and more accurately with relatively less hunting iterations.
**Step (2):** at each iteration ${\left\{t\right\}}_{t=1}^{T_{H}}$ over the hunt duration $T_{H}$, $-F_{c}\left(\Sigma \right)$ is evaluated over each individual $\Sigma_{t-1}^{\left(r\right)}$ in the pack, and the fittest three that better minimize it are identified as $\Sigma_{t-1}^{\alpha }$, $\Sigma_{t-1}^{\beta }$, and $\Sigma_{t-1}^{\delta }$, respectively.
**Step (3):** the so-called convergence factor $a_{t}$ guiding the hunt is updated according to one of the following cases (a) or (b).
**Step (3.a) [GWO or IS-GWO]:** $a_{t}=2\;\left(1-\frac{t}{T_{H}}\right)$ is simply set to decrease linearly from 2 to 0 over the hunt duration $T_{H}$. Therefore, the positions of the wolves to converge to local minima.
**Step (3.b) [GWOEIS]:** To improve and speed up convergence, instead of a common convergence factor, each realization or individual in the pack is assigned one of its own that accounts both for the AoA and TD pseudo-CDFs calculated in Steps (4) and (7) of the IS technique (cf. Section 3.2) as follows:
  (31) $$a_{j,t}^{\left(r\right)}=\frac{u_{j}^{\left(r\right)}}{t},$$
  where $u_{j}^{\left(r\right)}∼U\left([\epsilon_{j}^{\left(r\right)},\varrho_{j}^{\left(r\right)}]\right)$, j={1,2,⋯,2Q} is the dimension index, and
  (32) $$\epsilon_{j}^{\left(r\right)}=\left\{\begin{array}{c} \max\;\left\{\;J_{\theta_{j}|\tau_{j}}{\left(\Sigma_{t}^{\left(r\right)}\right)}_{j},J_{\theta_{j}|\tau_{j}}\left({\hat{\theta }}_{j}^{0}\right)\;\right\},\;\;j\;=\;\{\;1,\cdots \;,Q\;\}\\ \max\;\left\{\;J_{\tau_{j^{\prime }}}{\left(\Sigma_{t}^{\left(r\right)}\right)}_{j^{\prime }},J_{\tau_{j^{\prime }}}\left({\hat{\tau }}_{j^{\prime }}^{0}\right)\;\right\},\;\;j\;\;=\;\;\{\;Q\;+\;1\;,\cdots \;,2Q\;\} \end{array}\right.$$
  (33) $$\varrho_{j}^{\left(r\right)}\;=\;\left\{\begin{array}{c} \min\;\left\{\;J_{\theta_{j}|\tau_{j}}{\left(\Sigma_{t}^{\left(r\right)}\right)}_{j},J_{\theta_{j}|\tau_{j}}\left({\hat{\theta }}_{j}^{0}\right)\;\right\},\;\;j=\{\;1,\cdots \;,Q\;\}\\ \min\;\left\{\;J_{\tau_{j^{\prime }}}{\left(\Sigma_{t}^{\left(r\right)}\right)}_{j^{\prime }},J_{\tau_{j^{\prime }}}\left({\hat{\tau }}_{j^{\prime }}^{0}\right)\;\right\},\;\;j=\{\;Q+1\;,\cdots \;,2Q\;\} \end{array}\right.$$
  where ${\left(S\right)}_{i}$ denotes the *i*-th element of the set S. ${\hat{\tau }}_{q}^{0}$ and ${\hat{\theta }}_{q}^{0}$ are the initial TD and AoA IS estimates obtained in (20) and (25), respectively, and $j^{\prime }=j-Q$.

When using the linearly decreasing factor in Step (3.a), the positions of the wolves can converge to local minima. To mitigate this issue, we generate for each individual a specific convergence factor that decreases with the iteration index while accounting through the pseud-CDF values for the distance between the gray wolves and the prey. As long as a wolf is far away from the prey, the uniform variable will generate realizations closer to 1. Once this wolf gets closer to the prey, the realization becomes quasi-static since, the convergence factor is then mainly scaled by 1/t.

**Step (4):** For each lead gray wolf ∗=α, β, or δ, we generate two random values, $b_{j}^{*}$ and $d_{j}^{*},$ in $U\left([0,1]\right)$ for the calculation of two update coefficients $g_{j,t}^{*}$ (or $g_{j,t}^{*,(r)}$ with respect to each realization or individual *r* in the pack), and $c_{j}^{*}$ according to one of the following cases (a) or (b).

**Step (4.a) [GWO or IS-GWO]:**
(34) $$g_{j,t}^{*}=2\;a_{t}\;b_{j}^{*}-a_{t},\;\;\;\mathrm{and}\;\;\;c_{j}^{*}=2\;d_{j}^{*}.$$

In other words, the update coefficients $g_{j,t}^{*}$ and $c_{j}^{*}$ are assigned random values in $\left[-a_{t},a_{t}\right]$ and [0,2], respectively.

**Step (4.b) [GWOEIS]:**
(35) $$g_{j,t}^{*,(r)}=2\;a_{j,t}^{\left(r\right)}\;b_{j}^{*}-a_{j,t}^{\left(r\right)},\;\;\;\mathrm{and}\;\;\;c_{j}^{*}=2\;d_{j}^{*}.$$

Here, $g_{j,t}^{*,(r)}$ is assigned a random value in $\left[-a_{j,t}^{\left(r\right)},a_{j,t}^{\left(r\right)}\right]$.

**Step (5):** Let $\mathrm{dist}\left(*,\left(r\right),j\right)=|c_{j}^{*}{\left(\Sigma_{t-1}^{*}\right)}_{j}-{\left(\Sigma_{t-1}^{\left(r\right)}\right)}_{j}\;|$ denote the distance between the lead wolf * and the *r*th individual in the pack (or search agent) across the *j*-th dimension. The lead wolves’ positions are then updated with respect to each realization *r* in the pack through intermediate variables $\chi_{*}^{\left(r\right)}$ according to one of the following cases (a) or (b).

**Step (5.a) [GWO or IS-GWO]:**
(36) $${\left(\chi_{*}^{\left(r\right)}\right)}_{j}={\left(\Sigma_{t-1}^{*}\right)}_{j}-g_{j,t}^{*}\mathrm{dist}\left(*,\left(r\right),j\right).$$

**Step (5.b) [GWOEIS]:**
(37) $${\left(\chi_{*}^{\left(r\right)}\right)}_{j}={\left(\Sigma_{t-1}^{*}\right)}_{j}-g_{j,t}^{*,(r)}\mathrm{dist}\left(*,\left(r\right),j\right).$$

Hence, before coming back to Step (2) if $t<T_{H}$, each individual’s location is updated in either case based on these intermediate variables $\chi_{*}^{\left(r\right)}$ as follows:

(38) $${\left(\Sigma_{t}^{\left(r\right)}\right)}_{j}=\frac{{\left(\chi_{\alpha }^{\left(r\right)}\right)}_{j}+{\left(\chi_{\beta }^{\left(r\right)}\right)}_{j}+{\left(\chi_{\delta }^{\left(r\right)}\right)}_{j}}{3}.$$

**Figure 1:**
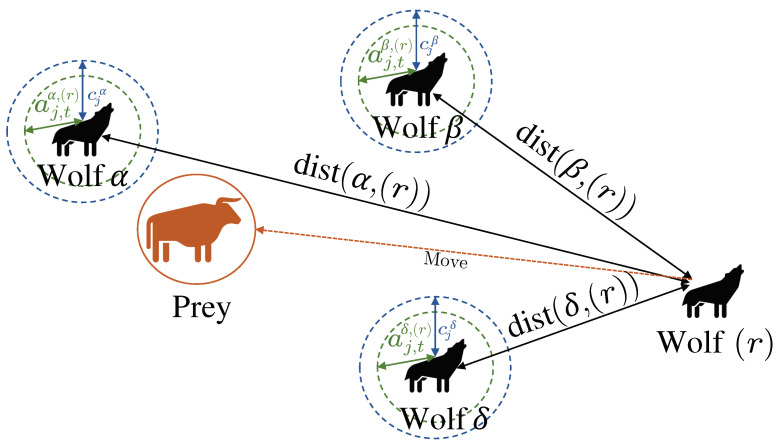
Position updating in GWOEIS.

**Figure 2:**
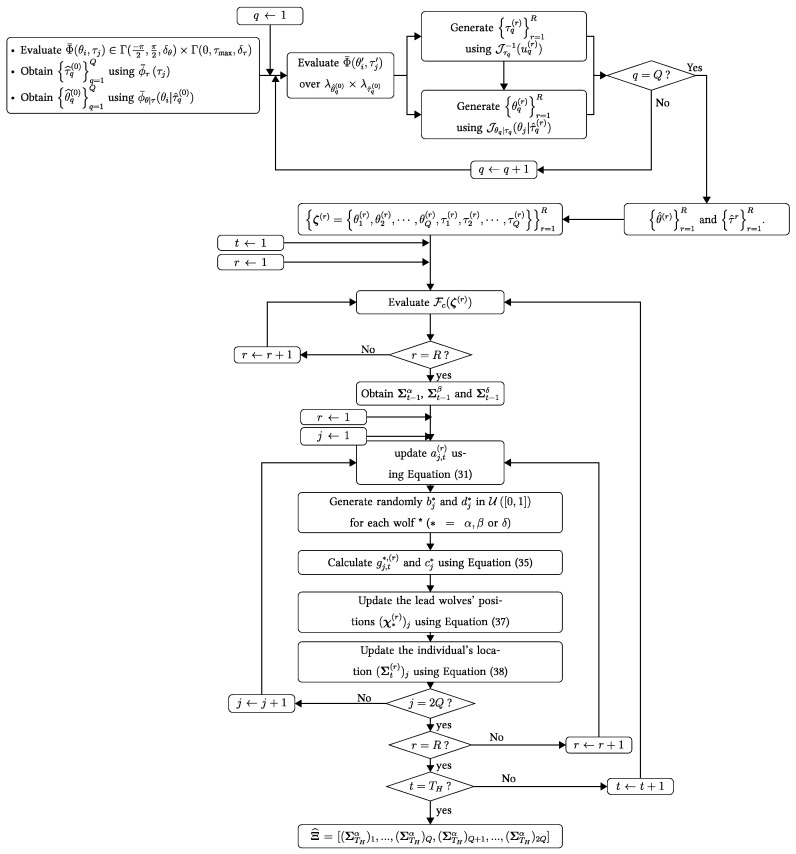
Flow chart of GWOEIS algorithm.

Figure 1 depicts the way to update a search agent’s position according to α-type, β-type, and δ-type wolves based on (37) and (38) for the proposed GWOEIS algorithm. Finally, the joint AoA-TD estimate ${\hat{\Xi }}_{\mathrm{Tech}}=\left[{\hat{\theta }}_{\mathrm{Tech}},{\hat{\tau }}_{\mathrm{Tech}}\right]$ is selected as the last position of the lead wolf α:

(39) $$\begin{array}{c} \;\;{\hat{\Xi }}_{\mathrm{Tech}}\;=\;\left[{\left(\Sigma_{T_{H}}^{\alpha }\right)}_{1},\ldots ,{\left(\Sigma_{T_{H}}^{\alpha }\right)}_{Q},{\left(\Sigma_{T_{H}}^{\alpha }\right)}_{Q+1},\ldots ,{\left(\Sigma_{T_{H}}^{\alpha }\right)}_{2Q}\right], \end{array}$$

where the choice of Tech ∈ {“GW0”, “IS-GWO”, “GWOEIS”} determines the cases considered in Steps (1), (3), (4), and (5). We summarize the GWOEIS algorithm in Figure 2.

## 4. Simulation Results

In this section, we assess the performance of the proposed GWOEIS solution and other key benchmarks for comparisons in terms of the root mean square error (RMSE) or the normalized MSE (NMSE) over a total number of Monte-Carlo runs $M_{c}=1000$. Following the IEEE 802.11ac standard (see [7] and first reference therein), we consider a bandwidth B=80 MHz with a sub-carrier spacing $\Delta_{f}=312.5$ KHz giving a total of 256 sub-carriers, among which 11 are exploited for network signaling purposes and the remainder are payload carriers (i.e., K=122). We also consider P=6 antennas and Q=2 equi-powered paths with AoAs $20^{\circ }$ and $45^{\circ }$ and TDs 25 ns and 62.5 ns, respectively. Moreover, we set $\rho_{1}=4$, $\delta_{\tau }=1.25$ ns, $\delta_{\theta }=0.1^{\circ }$, $\Delta_{\tau }=18.75$ ns, and $\Delta_{\theta }=10^{\circ }$.

**Figure 3:**
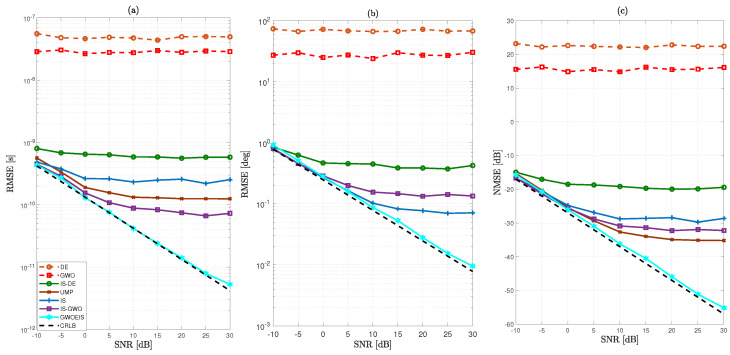
MSE vs. the SNR in dB for Q=2 ($\theta =[20^{\circ },\;45^{\circ }]$; τ = [25 ns, 62.5 ns]), R=30, and $T_{H}=100$ of: (**a**) the *Q* TDs, (**b**) the *Q* AoAs, and (**c**) the P×K channel coefficients (on average, per element, for all three parameter types).

**Figure 4:**
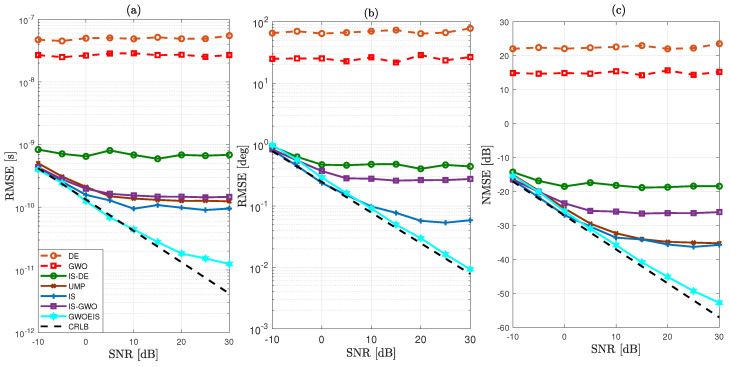
MSE vs. the SNR in dB for Q=2 ($\theta =[20^{\circ },\;45^{\circ }]$; τ = [25 ns, 62.5 ns]), R=100, and $T_{H}=30$ of: (**a**) the *Q* TDs, (**b**) the *Q* AoAs, and (**c**) the P×K channel coefficients (on average, per element, for all three parameter types).

In Figure 3 and Figure 4, we evaluate the RMSE/NMSE performance versus the SNR to compare the new GWOEIS solution against the Cramér–Rao lower bound (CRLB) of [6], the UMP algorithm in [7], the classic GWO in [28], the classic DE [13], the IS technique in [11], IS-DE that simply combines DE with IS in [13], and another benchmark version developed here by simply combining this time IS with GWO, referred to as IS-GWO.

As shown in Figure 3 and Figure 4, our approach outperforms all other estimation techniques, both in terms of TD and AoA estimations. Additionally, it reaches the CRLB, even at low SNR levels and even with a very low value either parameter R=30 and $T_{H}=30$. We also observe a severe performance degradation of the original GWO and DE techniques due to the high dimension (i.e., 2Q) of the optimization problem. When combined with IS, IS-DE improves a little but is outperformed by IS-GWO, and more so by GWOEIS.

In Figure 3c and Figure 4c, we compare the channel NMSE using the estimates of the TDs and AoAs assuming a perfect knowledge of the channel gains. Our approach remarkably outperforms all other estimation techniques and reaches the lower bound, even at very low SNR levels and even with a very low value either parameter R=30 and $T_{H}=30$.

**Figure 5:**
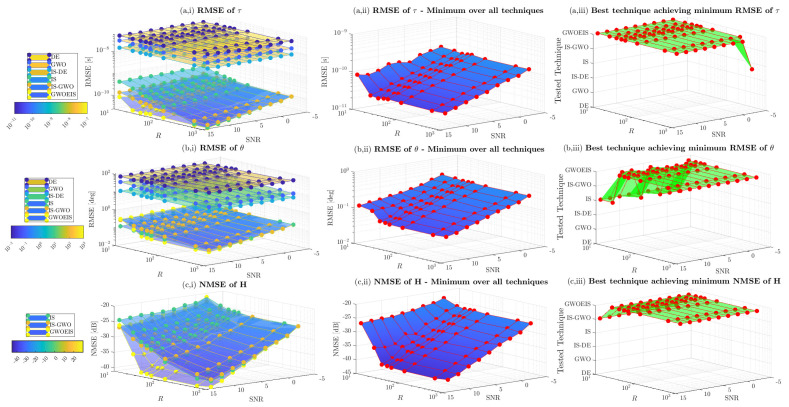
MSE vs. the SNR in dB and the samples size *R* for Q=2 ($\theta =[20^{\circ },\;45^{\circ }]$; τ = [25 ns, 62.5 ns]) and $T_{H}=100$.

**Figure 6:**
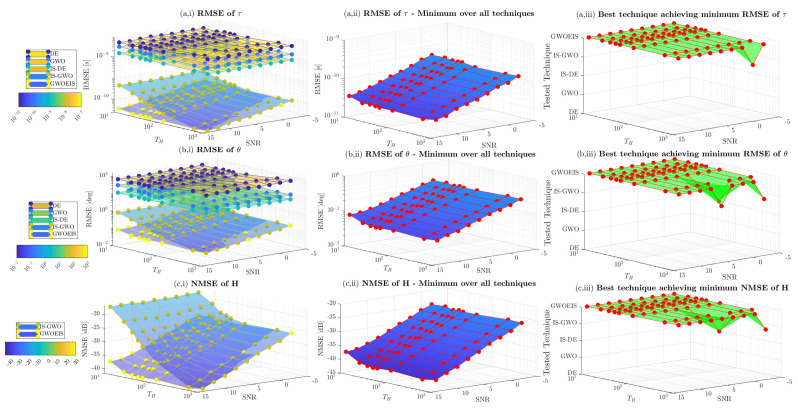
MSE vs. the SNR in dB and the iterations number (e.g., $T_{H}$) for Q=2 ($\theta =[20^{\circ },\;45^{\circ }]$; τ = [25 ns, 62.5 ns]) and R=100.

In Figure 5 and Figure 6, we assess the impact of the samples size *R* and the hunting duration $T_{H}$ on channel estimation performance versus the SNR. Here, the sample size *R* refers to the number of realizations with IS, to the wolves pack size with GWO, IS-GWO, and GWOEIS, or the number of individuals with DE and IS-DE. The number of iterations (e.g., $T_{H}$) is defined only for iterative approaches, i.e., GWO, IS-GWO, GOWEIS, DE, and IS-DE solutions. We notice that both parameters can have a detrimental effect on the exploration abilities of these techniques. Thus, they need to be set as large as possible to cover the widest area of the multidimensional space.

In Figure 5(a,i,b,i,c,i), we assess the estimation performance of the TD, the AoA, and the channel, respectively. GWOEIS, IS-GWO, and IS performs better than DE, GWO, and IS-DE. Figure 5(a,ii,b,ii,c,ii) show the minimum RMSE over all techniques for TD, AoA, and channel estimation, respectively. The RMSE decreases when *R* and SNR increase. In Figure 5(a,iii,b,iii,c,iii), we can see that GWOEIS achieves the best performance in terms of TD, AoA, and channel estimation over R≥20 and all SNR values. IS, IS-GWO, GWO, DE, and IS-DE can not match the performance of GWOEIS, even with R=1000, but at the expense of significant increase in computational cost.

In Figure 6, we assess the impact of $T_{H}$ on RMSE performance of the iterative techniques. Once again, GWOEIS outperforms all other iterative techniques in terms of TD, AoA, and channel estimation. By increasing the number of iterations, we reach the best performance for the all algorithms, as shown in Figure 6(a,ii,b,ii,c,ii). Moreover, in Figure 6(a,iii,b,iii,c,iii), we notice that a small order of 10 iterations is enough to put GWOEIS on the top of the rest, with estimation accuracy gains constantly increasing with $T_{H}$, making its potential gains in computational complexity remarkably large when compared to other techniques that require a very high number of iterations.

**Figure 7:**
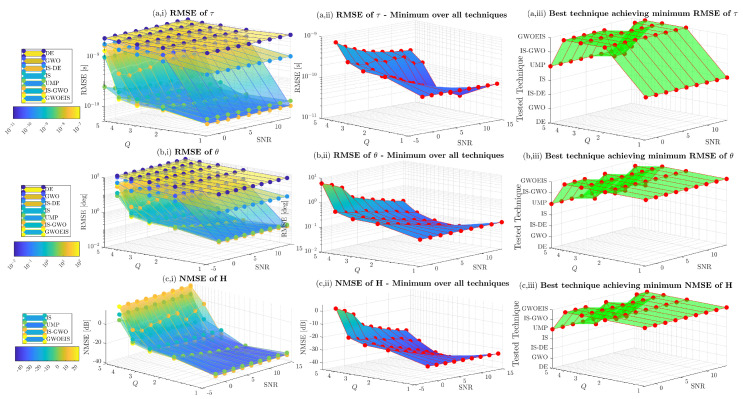
MSE vs. the SNR in dB and the number of paths *Q* for $T_{H}=100$ and R=100.

In Figure 7, we investigate the effect of the number of paths on the estimation performance. We observe that the new approach, GWOEIS, outperforms most of the benchmarks in the high and low SNR scenarios for TD, AoA, and channel estimation when Q≤4.

**Figure 8:**
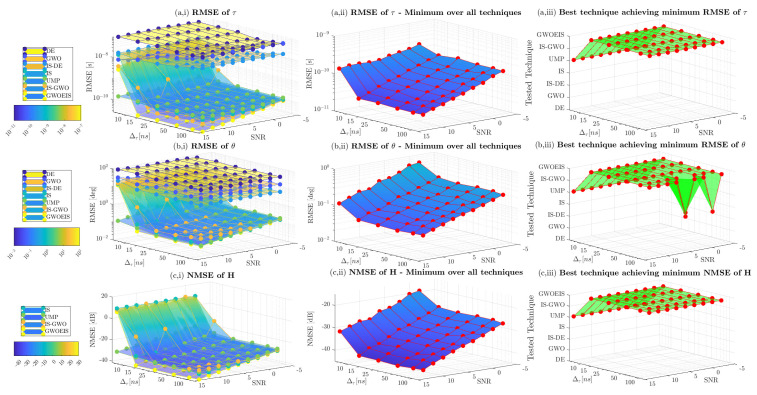
RMSE vs. the SNR in dB and the temporal separation $\Delta_{\tau }$ in ns with Q=2, R=100, and $T_{H}=100$.

**Figure 9:**
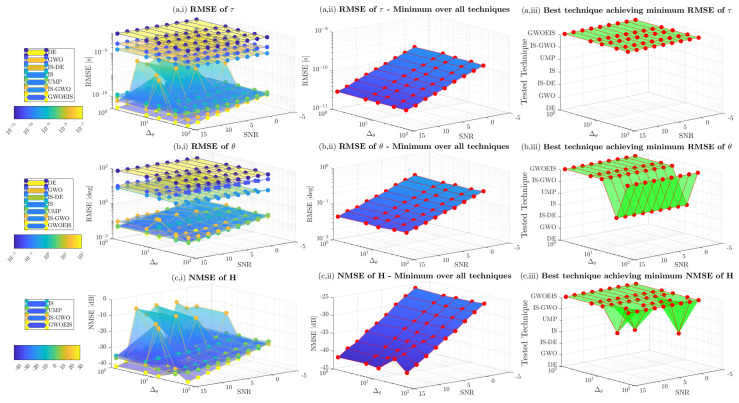
RMSE vs. the SNR in dB and the angular separation $\Delta_{\theta }$ in degrees with Q=2, R=100, and $T_{H}=100$.

We consider also the same configuration to evaluate the performance of all of the approaches in terms of temporal and angular separations. In Figure 8, we fix the first delay at $\tau_{1}=2\;T$, and we vary the second delay at $\tau_{2}=\tau_{1}+\Delta \tau$. It is clearly seen that our approach is still capable of achieving the CRLB, even in a challenging scenario with a very small temporal separations of Δτ=0.5T. In Figure 9, we fix the first AoA at $\theta_{1}=-20^{\circ }$, and we vary the second AoA $\theta_{2}=\theta_{1}+\Delta \theta$. Here, again, we can highlight the robustness and the super-resolution capacity of our approach, and to appreciate its superiority in challenging scenarios where the paths are closely spaced in both temporal and spatial domains. We have to mention that Figure 8(a,iii,b,iii,c,iii) show the superiority of UMP reaching the best techniques achieving minimum RMSE only when $\Delta_{\tau }\geq 10/B$.

## 5. Conclusions

In this work, we presented a new DA ML JADE estimator over OFDM SIMO multi-path channels referred to as GWOEIS, whose key innovation lies in exploiting and embedding the powerful IS concept to avoid the random initialization issues of the traditional GWO, and to significantly improve the hunting mechanism. GWOEIS ensures faster convergence by providing initial estimates based on a simplified importance function. More importantly, and beyond simple initialization of GWO with IS, we modify and dynamically update the conventional simple expression for the convergence factor of the GWO algorithm that entirely drives its hunting and tracking mechanism by accounting for new CDFs derived from the IS technique. The latter significantly boost the estimation performance. Overall, the simulation results show more accurate estimation performance at faster convergence rates with GWOEIS.

## Data Availability

Data are contained within the article.

## Abbreviations

The following abbreviations are used in this manuscript:

AoA	Angle of Arrival
AWGN	Additive White Gaussian Noise
CDF	Cumulative Distribution Function
CFR	Channel Frequency Response
CLF	Concentrated Likelihood Function
CRLB	Cramér–Rao Lower Bound
DA	Data-Aided
DE	Differential Evolution
DoA	Direction of Arrival
GWO	Gray Wolf Optimization
GWOEIS	Gray Wolf Optimization Embedding Importance Sampling
IS	Importance Sampling
IS-DE	Importance Sampling–Differential Evolution
IS-GWO	Importance Sampling–Gray Wolf Optimization
JADE	Joint Angle and Delay Estimation
LLF	Log-Likelihood Function
LS	Least Squares
ML	Maximum Likelihood
MSE	Mean Square Error
MUSIC	Multiple Signal Classification
NDA	Non-Data-Aided
NMSE	Normalized MSE
OFDM	Orthogonal Frequency-Division Multiplexing
PDF	Probability Density Function
RMSE	Root Mean Square Error
SAGE	Space-Alternating Generalized Expectation
SIMO	Single Input Multiple Output
SNR	Signal-to-Noise Ratio
TD	Time Delay
UAV	Unmanned Aerial Vehicles
UMP	Unitary Matrix Pencil
